# Pilot-Scale Hydrolysis-Aerobic Treatment for Actual Municipal Wastewater: Performance and Microbial Community Analysis

**DOI:** 10.3390/ijerph15030477

**Published:** 2018-03-09

**Authors:** Xiao Bian, Hui Gong, Kaijun Wang

**Affiliations:** State Key Joint Laboratory of Environment Simulation and Pollution Control, School of Environment, Tsinghua University, Beijing 100084, China; bianxiao8@126.com

**Keywords:** hydrolysis-aerobic, municipal wastewater treatment, microbial community, high-throughput sequencing

## Abstract

Low-energy cost wastewater treatment is required to change its current energy-intensive status. Although promising, the direct anaerobic digestion of municipal wastewater treatment faces challenges such as low organic content and low temperature, which require further development. The hydrolysis-aerobic system investigated in this study utilized the two well-proven processes of hydrolysis and aerobic oxidation. These have the advantages of efficient COD removal and biodegradability improvement with limited energy cost due to their avoidance of aeration. A pilot-scale hydrolysis-aerobic system was built for performance evaluation with actual municipal wastewater as feed. Results indicated that as high as 39–47% COD removal was achieved with a maximum COD load of 1.10 kg/m^3^·d. The dominant bacteria phyla included *Proteobacteria* (36.0%), *Planctomycetes* (15.4%), *Chloroflexi* (9.7%), *Bacteroidetes* (7.7%), *Firmicutes* (4.4%), *Acidobacteria* (2.5%), *Actinobacteria* (1.8%) and *Synergistetes* (1.3%), while the dominant genera included *Thauera* (3.42%) and *Dechloromonas* (3.04%). The absence of methanogens indicates that the microbial community was perfectly retained in the hydrolysis stage instead of in the methane-producing stage.

## 1. Introduction

Wastewater treatment accounts for a large amount of the energy load in society as a whole. It is estimated that 3–4% of total U.S. electricity is consumed for the movement and treatment of water and wastewater [1]. A similar situation could be observed in other developed and developing countries. High energy consumption also creates a large carbon footprint, accelerating global warming. As wastewater production increases due to population and economic growth, the energy-water burden becomes heavier for the future, undermining social sustainability. Thus, it is of great importance to reduce energy costs during the treatment of wastewater. 

Wastewater treatment, which is mainly based on the conventional active sludge process (CAS), is criticized as energy-intensive for its heavy aeration. Based on a full-scale plant energy audit, energy as high as 50% was used to supply air for aeration tanks during the CAS process. The anaerobic digestion (AD) process, which avoids aeration and meanwhile recovers energy in the form of CH_4_ from organics, is a perfect alternative choice for wastewater treatment [2]. Based on energetic calculation, the energy potential in domestic wastewater is higher than that consumed during treatment [3]. Namely, it is even possible to turn wastewater treatment (WWTP) into a net energy producer using a carefully designed process [4]. However, full-scale direct anaerobic treatment of domestic wastewater is mainly applied in developing countries in (sub)tropical regions such as Brazil, Mexico, Egypt, and India. Its performance is generally unsatisfactory. Challenges exist for its wide application, including a low concentration of organics and low temperature in winter in temperate regions, both of which usually lead to biomass loss, inefficient organic biodegradation, and poor effluent quality. 

Multiple measures have been considered to solve this problem, such as enhancing the anaerobic methane-producing process. The most viable idea is the use of the anaerobic membrane bioreactor (AnMBR), which has been thoroughly investigated in past decades [5]. The AnMBR utilizes the membrane with a high separation efficiency to retain enough biomass to maintain a sufficiently high solids retention time (SRT). The quality of effluent is also guaranteed with the use of membrane filtration. Recently another idea, which has drawn much attention, is to separate and concentrate organics in limited volume. First, this is performed through coagulation, adsorption or membrane filtration, and then it anaerobically transforms the concentrated organics into CH_4_ in a more conventional way for energy recovery [6]. Although all these developments are promising, further efforts are still needed for their scale-up and full-scale application. 

The hydrolysis-aerobic hybrid treatment investigated in this study combined the two well-proven processes of hydrolysis and aerobic oxidation, which was expected to achieve the advantages of the two processes. Due to the limitations of anaerobic methane production mentioned above, part of AD—namely hydrolysis—was applied and combined with aerobic post-treatment. Based on the individual bacteria species, the anaerobic process can be divided into three stages, including hydrolysis, acidogenesis and methanogenesis [7]. Hydrolysis is the initial stage when complex organic polymers are hydrolyzed into simple molecules by hydrolytic enzymes of fermenting bacteria. During the acidogenesis and methanogenesis processes, intermediate products including volatile fatty acids (VFA), such as acetate and H_2_, were generated and finally converted into CH_4_. For the first stage of AD, hydrolysis has been widely used as a biological pre-treatment. Hydrolysis can not only partially remove COD by fermentation and maybe also respiration of some of the hydrolysis products, but biodegradability can also be improved. Hydrolysis has been used for a lot of refractory feed, including both solid and liquid waste such as lignocellulosic biomass (e.g., sugarcane bagasse and rice straw) [8], excess sludge [9], food waste [10] and refractory/particulate-rich wastewater from tanneries [11] and the petrochemical industry [12]. The inhibiting issue of the antibiotic (e.g., tetracycline) production of wastewater for the following biological process could be avoided through the use of hydrolysis with a BOD/COD ratio increase [13]. Moreover, hydrolysis is based on anaerobes with no aeration and no additional heating requirements, indicating its low-energy cost for large-volume municipal wastewater treatment. Combined with aerobic post-treatment, effluent quality could be improved and also the energy requirement for post-aeration could be reduced due to a lower COD load and high biodegradability. Therefore, the hydrolysis-aerobic hybrid treatment process was expected to be a potential low-cost approach for the treatment of municipal wastewater. However, the performance of the hydrolysis-aerobic process still requires optimization through an investigation on the effects of operation parameters such as the COD load. The structure of the bacterial community also needs to be revealed.

In this study, a pilot-scale hydrolysis-aerobic system was built for performance evaluation with actual municipal wastewater as feed. The influence of the COD load on hydrolysis was investigated. The bacterial community structure of the hydrolysis system was analyzed using high-throughput sequencing.

## 2. Materials and Methods 

### 2.1. Pilot Plant Experiments

**Figure 1:**
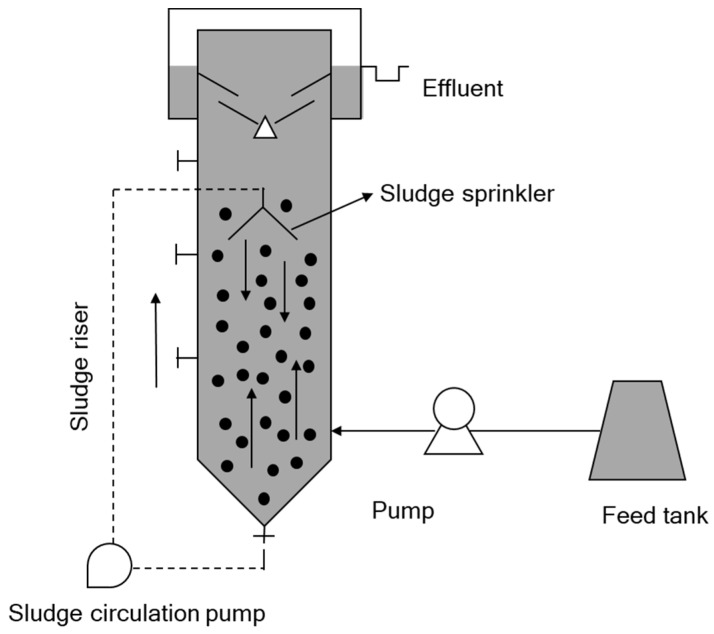
A schematic view of the hydrolysis reactor.

The performance of hydrolysis-aerobic treatment was evaluated using a pilot-scale hydrolysis reactor (Figure 1) and aerobic post-treatment. All the pilot facilities were installed in Caoxian WWTP in Shandong province, China. Pilot-scale hydrolysis reactor with a vertical acrylic column (2 m diameter × 6 m length) was used, with double loop inside. It was composed of four main parts: the cone on the bottom, the cylinder, the tri-phase separator and the sludge circulation system. The sludge circulation pump controlled the rate of internal sludge circulation and provided suitable shear force for sludge at different growth stages. Sludge flowed from top to the bottom and sewage moved up-flow, with the down-flow/up-flow ratio more than 10:1 at the start-up stage. 

Actual municipal wastewater after the coarse filter of Caoxian WWTP was taken as feed. The BOD/COD of municipal wastewater feed was as low as about 0.2, due to some industrial wastewater flowing into WWTP. Sludge from the thickening tank was inoculated into the hydrolysis reactor with a final concentration of 25 g/L. The effective volume of the hydrolysis reactor is 16.5 m^3^. Pilot-scale experiments lasted for approximately 45 days and had four stages. During the pilot experiment, temperature was maintained around 25 °C. The first stage was a start-up with approximately 50 h of hydraulic retention time (HRT). After 16 days of operation, HRT was reduced to 21 and 12 h in stage II and stage III with COD increasing, respectively. To evaluate the limitation, HRT was reduced further to 5 h in stage IV until the operation performance collapsed. 

### 2.2. Analytical Methods

The chemical oxygen demand (COD) and NH_4_-N were determined using colourimetric techniques with a HACH spectrophotometer (DR 5000, HACH, Loveland, CO, USA). Illumina HiSeq (Santiago, MN, USA) sequencing was applied to analyze the microbial consortium of the last stage sludge from the hydrolysis reactor. The procedures used are as follows: (1) The total genomic DNA was extracted using a DNA extraction kit (Mo Bio Laboratories, Carlsbad, CA, USA) according to the manufacturer’s instructions; (2) PCR amplification was performed using specifically synthesized primers with a barcode (Forward: GTGCCAGCMGCCGCGGTAA, Reverse: GGACTACHVGGGTWTCTAAT) in the ABI GeneAmp^®^ 9700 system (Bio-Rad, Hercules, CA, USA) using the following programme: 5 min of denaturation at 95 °C followed by 30 cycles of 30 s at 95 °C (denaturation), 30 s for annealing at 58 °C and 25 s at 72 °C (elongation), with a final extension at 72 °C for 7 min, and polymerase chain reaction (PCR) products were saved for Illumina HiSeq sequencing; and (3) high-throughput sequencing was performed using the Illumina HiSeq 2500 platform (Illmumina, Santiago, MN, USA). 

## 3. Results and Discussion

### 3.1. Effects of the Organic Loading Rate (OLR) on Hydrolysis 

As shown in Figure 1, the performance of a pilot-scale hydrolysis reactor was evaluated from start-up to stable stages with an increasing COD load. Stage I was a start-up with an average COD load of 0.14 kg/m^3^·d. At the start, there was little to even negative COD removal due to inoculum sludge cell lysis and the release of organics. After 8 days of acclimatization, hydrolysis took over the main reaction and COD removal reached 40~50%. At the start-up stage, the total average COD removal was 25.1%. During stages II and III, the COD load increased to 0.34 kg/m^3^·d and 0.71 kg/m^3^·d. Meanwhile, the COD removal also increased to averages of 39.3% and 47.7%. The influence and effluence COD during pilot experiments is shown in Table 1. It should be noted that although the average COD removal efficiencies increased comparing Phases II and III, it was not significantly different due to fluctuating influent COD and limited measurements.

In the last stage, stage IV, the COD load increased to 1.10 kg/m^3^·d with a very short HRT of 4.97 h. It should be noted that the hydrolysis performance was negatively impacted in this condition and the COD removal was only 20.2%. The main reason for the collapse of COD removal is probably the loss of microbial sludge in hydrolysis reactor due to the short HRT and high up-flow velocity. Recent research reporting granular hydrolysis sludge with much higher settleability was potential solution to improve performance of hydrolysis reactor, which could be investigated in further research.

### 3.2. Performance of the Hydrolysis-Aerobic System

**Figure 2:**
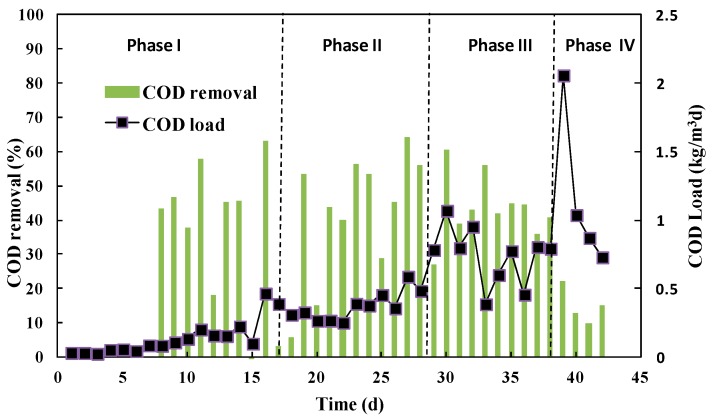
Effects of the organic loading rate (OLR) on the COD removal (based on TCOD) of the hydrolysis reactor.

The performance of the hydrolysis-aerobic system (stages III and IV) is illustrated in Figure 2. The COD of influent, after hydrolysis and the final effluent during stage III, was 244.3 ± 70.7 mg/L, 127.8 ± 40.4 mg/L and 59.6 ± 14.1 mg/L. The removal efficiencies of hydrolysis and the total hydrolysis-aerobic system were 44.8 ± 9.7% and 71.9 ± 13.1%, respectively. As high as 44.8% COD removal was achieved during hydrolysis, indicating its high efficiency. Additionally, the fluctuation was reduced for COD after hydrolysis, indicating its anti-shock loading capability. In other words, the hydrolysis system is a pre-treatment process with an anti-shock loading capability. The performance of the aeration process was not optimized and the effluent COD was still higher than 50 mg/L. It is expected that effluent COD can be reduced through modified aeration. 

In comparison, at stage IV after hydrolysis and the final effluent, the COD of influent was 242.0 ± 122.8 mg/L, 201.5 ± 88.4 mg/L and 48.0 ± 8.2 mg/L. The removal ratios of hydrolysis and the total hydrolysis-aerobic system were 15.1 ± 5.1% and 75.8 ± 12.0%, respectively. The COD removal achieved through hydrolysis was remarkably limited due to the high COD load and the very short HRT over-reactor limits. Although the total COD removal of stage IV is close to that of stage III, its aeration energy consumption was probably higher than that of stage III. Furthermore, little nitrogen was removed by hydrolysis. No obvious NH_4_-N reduction was observed (20.3 ± 4.8 mg/L after hydrolysis with influent of 19.3 ± 6.1 mg/L). Nitrification occurred in subsequent aeration process and NH_4_-N decreased to 2.9 ± 1.0 mg/L in final effluent.

From the perspective of cost saving, the hydrolysis-aeration hybrid system demonstrates its potentials on two advantages. The first is energy saving by degrading part of organics during hydrolysis process, which otherwise would be removal by energy-intensive aeration process e.g., about 105~120 mg/L COD was removal during hydrolysis (stages II and III). Here we assume the energy for aeration was providing 1 kg O_2_/kWh energy input. Based the roughly estimation, the energy saving by COD removal of hydrolysis process could be as high as 0.11~0.12 kWh/m^3^, indicating its advantage over conventional process. The other potential advantage is to improve biodegradability by degrade complex organic compounds into small molecules during hydrolysis.

### 3.3. Microbial Community Analysis

**Figure 3:**
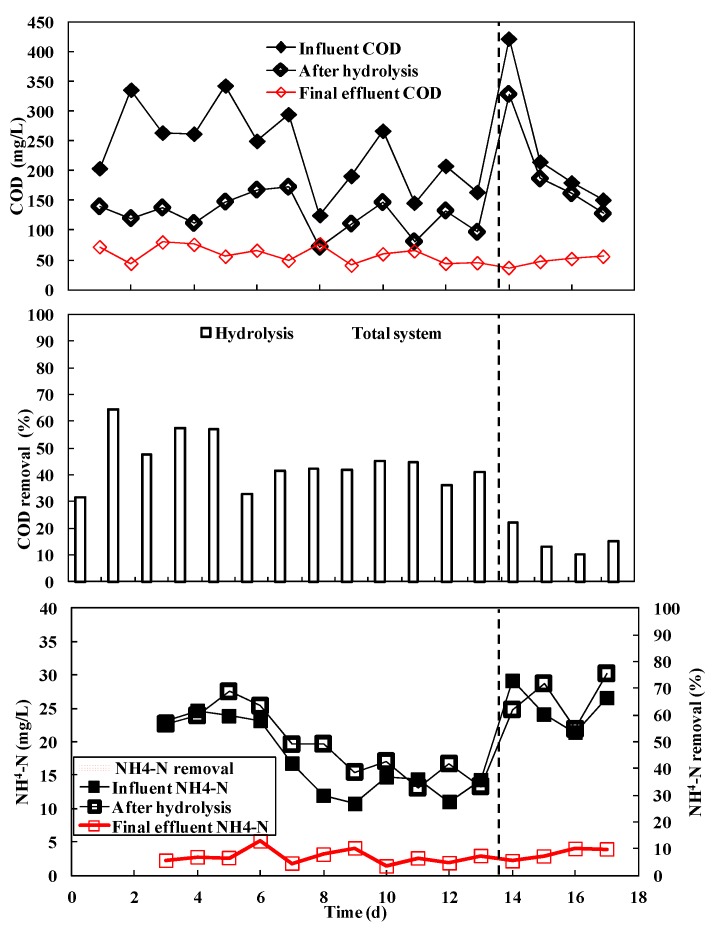
COD and NH_4_^+^-N concentration of the influent and effluent and their removal efficiencies during the hydrolysis-aerobic process (stages III and IV).

The structure and diversity of bacterial communities in the hydrolysis reactor were revealed using high-throughput sequencing (HiSeq). Analysis of both the phylum and genus levels of the bacterial community is shown in Figure 3. Bacteria phyla with a relative abundance above 1% included *Proteobacteria* (36.0%), *Planctomycetes* (15.4%), *Chloroflexi* (9.7%), *Bacteroidetes* (7.7%), *Firmicutes* (4.4%), *Acidobacteria* (2.5%), *Actinobacteria* (1.8%) and *Synergistetes* (1.3%). These phyla were also identified by other researchers as the dominant bacteria phyla for wastewater treatment [14]. It is reported that Proteobacteria, Bacteroidetes and Firmicutes are dominant bacteria phyla in a conventional active sludge process and also in a hydrolysis system. In this study, they account for approximately 48.1%. Proteobacteria has been widely reported in the wastewater treatment process for its multiple functions [15]. It was revealed that Bacteroidetes has the capacity to convert organic carbon to CO_2_ through aerobic oxidation [16]. Firmicutes, as reported previously, was connected with hydrolyzing long-chain organics into small molecules [17]. The hydrolysis reactor exhibited a strong performance in removing and degrading the complex organic compounds in the municipal wastewater.

**Figure 4:**
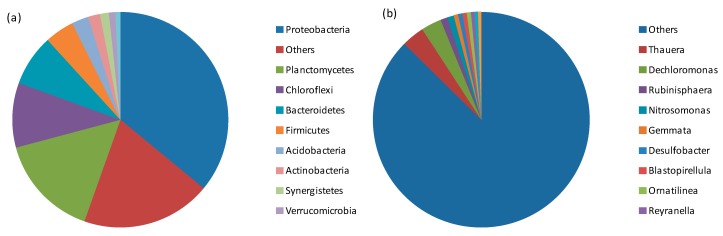
Microbial community structures in the hydrolysis reactor at phylum level (**a**) and genus level (**b**).

The hydrolysis processes required the presence of a diverse group of bacteria, which were probably closely dependent on each other. There were as many as approximately 200 genera identified in the hydrolysis system. The utilisation of actual wastewater increased the complexity of the microbial niche and led to high diversity. As high as 87.4% of the bacteria were unclassified and even unknown based on genus-level analysis. As shown in Figure 4b, the top ten genera included *Thauera* (3.42%), *Dechloromonas* (3.04%), *Rubinisphaera* (1.05%), *Nitrosomonas* (0.98%), *Gemmata* (0.66%), *Desulfobacter* (0.65%), *Blastopirellula* (0.64%), *Ornatilinea* (0.63%), *Reyranella* (0.56%), *Thiobacillus* (0.48%), *Longilinea* (0.48%). The genus *Thauera* belongs to the *Betaproteobacteria* class and the Proteobacteria phylum. It had a denitrification capacity and is widely distributed in wastewater treatment plants, rivers and polluted pools [18]. It is also reported to be dominant in the denitrification process, such as in the DEAMOX (Denitrifying Ammonium OXidation) system. *Dechloromonas* frequently demonstrated a capacity to degrade aromatic compounds such as benzene, and this was related to nitrogen removal [19]. Furthermore, it is widely found in WWTP effluence [19]. *Nitrosomonas* was strictly of the ammonia-oxidizing bacterial (AOB), indicating the potential for nitrosification [20]. *Desulfobacter* is a genus of sulfate-reducing bacteria (SRB) that utilise sulfate as an electron accepter and degrade aromatic compounds. Together with *Desulfobacula*, it was classified as part of the Proteobacteria phylum, *Deltaproteobacteria* class, *Desulfobacterales* order and *Desulfobacteraceae* family. During hydrolysis, no aeration reduced the SO_4_^2−^ reduction and inhibited SRB abundance and diversity [12]. *Longilinea* of the *Anaerolineae* class and *Thiobacillus* of the *Betaproteobacteria* class were reported to degrade refractory organics including naphthaline and SCN^−^.

Archaeal diversities and communities were also investigated. No methanogens were found as dominant and only *Methanomassiliicoccus* was identified with a very low abundance of 0.0096%. Both the absence of methanogens and the process performances indicated that the microbial community was perfectly retained within the hydrolysis stage instead of entering into the methane-producing stage. Moreover, it should be noted that microbial community structure analysis by HiSeq was performed only for the last stage with single sampling. The evolving of microbial community structure could be revealed by further research with various sampling over different stages and it would be more informative approach to provide microbial information. 

## 4. Conclusions

The hydrolysis achieved effective pre-treatment with 39–47% COD removal in the hybrid hydrolysis-aerobic process for the low-cost treatment of municipal wastewater. The performance of the pilot-scale system was inhibited when the COD load increased to 1.10 kg/m^3^·d. The microbial community in the hydrolysis reactor was analyzed. The dominant bacteria phyla included *Proteobacteria* (36.0%), *Planctomycetes* (15.4%), *Chloroflexi* (9.7%), *Bacteroidetes* (7.7%), *Firmicutes* (4.4%), *Acidobacteria* (2.5%), *Actinobacteria* (1.8%) and *Synergistetes* (1.3%). The dominant bacteria genera included *Thauera* (3.42%) and *Dechloromonas* (3.04%). High microbial diversity was observed with actual wastewater as feed, indicating a large proportion of genera that needs to be investigated further. The absence of methanogens indicates that the microbial community was perfectly retained in the hydrolysis stage instead of in the methane-producing stage.

## Figures and Tables

**Table 1:** COD removal during different stages of hydrolysis.

	Influent COD (mg/L)	Effluent COD (mg/L)	Removal Efficiency (%)	HRT (h)
Phase I	244.8 ± 138.4	183.4 ± 70.2	25.1	51.68
Phase II	270.3 ± 53.1	164.1 ± 44.4	39.3	21.29
Phase III	244.3 ± 70.7	127.8 ± 40.4	47.7	12.78
Phase IV	242.0 ± 122.8	201.5 ± 88.4	16.7	4.97
